# Identification of the differentially expressed genes associated with familial combined hyperlipidemia using bioinformatics analysis

**DOI:** 10.3892/mmr.2015.3263

**Published:** 2015-01-27

**Authors:** XIAOLI LUO, CHANGQING YU, CHUNJIANG FU, WEIBIN SHI, XUKAI WANG, CHUNYU ZENG, HONGYONG WANG

**Affiliations:** Department of Cardiology, Daping Hospital, The Third Military Medical University, Chongqing Institute of Cardiology, Chongqing 400042, P.R. China

**Keywords:** familial combined hyperlipidemia, differentially expressed genes, function enrichment analysis, protein-protein interaction network

## Abstract

The aim of the present study was to screen the differentially expressed genes (DEGs) associated with familial combined hyperlipidemia (FCHL) and examine the changing patterns. The transcription profile of GSE18965 was obtained from the NCBI Gene Expression Omnibus database, including 12 FCHL samples and 12 control specimens. The DEGs were identified using a linear models for microarray data package in the R programming language. Gene Ontology (GO) function and Kyoto Encyclopedia of Genes and Genomes (KEGG) pathway enrichment analysis was also performed. Protein-protein interaction (PPI) networks of the DEGs were constructed using the EnrichNet online tool. In addition, cluster analysis of the genes in networks was performed using ClusterONE. A total of 879 DEGs were screened, including 394 upregulated and 485 downregulated genes. Enrichment analysis identified four important KEGG pathways associated with FCHL: One carbon pool by folate, α-linolenic acid metabolism, asthma and the glycosphingolipid biosynthesis-globo series. GO annotation identified 12 enriched biological processes, including one associated with hematopoiesis and four associated with bone cell differentiation. This identification was in accordance with clinical data and experiments into hyperlipidemia and bone lesions. Based on PPI networks, these DEGs had a close association with immune responses, hormone responses and cytokine-cytokine receptors. In conclusion, these DEGs may be used as specific therapeutic molecular targets in the treatment of FCHL. The present findings may provide the basis for understanding the pathogenesis of FCHL in future studies. However, further experiments are required to confirm these results.

## Introduction

Familial combined hyperlipidemia (FCHL), the most common genetic form of hyperlipidemia, is characterized by significant familial clustering and premature coronary heart disease (1). FCHL is a common inherited disorder of lipid metabolism with a prevalence of 0.5–2.0%, accounting for 10% of the cases of premature coronary heart disease worldwide (2). Therefore, the research and treatment of FCHL has significance for human health. Multiple hyperlipemic phenotypes have been characterized in the same individual and in the same family, which can be detected by elevated very-low-density lipoproteins (VLDL) and low-density lipoproteins (LDL) or apolipoprotein B (apoB) (3,4).

To date, studies have focused on the molecular mechanisms of FCHL development in order to reveal biomarkers for clinical treatment. The FCHL locus has been mapped to human chromosome 1q21–q23. This region includes retinoid X receptor γ (RXRG), a nuclear factor member of the RXR superfamily, which is critical in lipid homeostasis (1). Sentinelli *et al* (1) have identified five polymorphisms in the RXRG gene (rs1128977, rs2651860, rs2134095, rs283696 and rs10918169). Hsieh *et al* (5) suggested that one single nucleotide polymorphism (SNP) in the RXRG gene, (rs3818569 now merged into rs1128977) has a positive correlation with the development of diabetic retinopathy. The rs2651860 SNP was significantly associated with increased levels of LDL-cholesterol and of apoB in T-allele carriers (1). A total of three SNPs in RXRÎ^3^ exhibited a significant association with HIV lipodystrophy (6).

In previous years, multiple candidate genes have been identified as associated with the FCHL phenotype. The upstream transcription factor 1 (USF1) is a transcription factor, which regulates the expression of a number of genes involved in glucose and lipid metabolism, and provides an adequate candidate for FCHL (7). Preliminary functional data suggested that the USF1 risk haplotype may affect the expression profiles in fat biopsy samples from individuals with FCHL (8). The lipoprotein lipase (LPL) gene is also a noteworthy candidate for FCHL. The decreased activity of LPL in subjects with FCHL has been identified and positive associations have been reported between FCHL and genetic variants in the LPL promoter and exon (9). In brief, these candidate gene studies may provide a theoretical foundation for FCHL treatment.

In the present study, the aim was to analyze the FCHL samples and control samples with a series of biological information technology services, with the purpose of revealing the mechanism underlying the development of FCHL. Gene-set enrichment analysis was performed and a protein-protein interaction (PPI) network was constructed. Functional genes and signaling pathways in FCHL were used to establish a theoretical foundation for future research. Present findings may provide a basis for understanding the pathogenesis of FCHL in the future.

## Materials and methods

### Data sources

The transcription profile of GSE1010 was obtained from the NCBI Gene Expression Omnibus (GEO) database (http://www.ncbi.nlm.nih.gov/geo/), which is based on the Affymetrix Human Genome U133A Array GPL96 (Affymetrix, Santa Clara, CA, USA). There were a total of 24 RNA specimens (lymphoblastic cells), including 12 FCHL samples and 12 control specimens.

### Screening of differentially expressed genes (DEGs)

The linear models for microarray data package (10) in the R programming language was used to identify DEGs. The original expression datasets were normalized using the normalize within arrays method and normalize between arrays method (11). Following normalization, the expression value was used to construct a linear model in order to identify the DEGs (12). P<0.05 was set as the cut-off criteria.

### Kyoto Encyclopedia of Genes and Genomes (KEGG) pathway analysis based on the PPI network

The KEGG pathway enrichment analysis of DEGs was performed using EnrichNet (http://www.enrichnet.org/) (13). EnrichNet is an analysis approach based on the PPI networks. EnrichNet calculates the overlap between the known KEGG pathways and constructed PPI networks, in order to acquire the PPI enriched KEGG pathway. In the present study, PPI networks of DGEs were constructed via the Search tool for the retrieval of interacting genes/proteins (STRING; http://www.string-db.org/) (14) database and the similarity between the PPI networks and the KEGG pathways were calculated via EnrichNet. The similarity was presented as an XD-score. The higher the XD-score value, the higher the similarity is, indicating an increased possibility of a KEGG pathway enriched with DEGs. In order to notarize the criteria of the XD-score, the classical overlap-based Fisher test was used to calculate the significance score (q-value) via EnrichNet and linear regression analysis between the q-value and XD-score was performed. An XD-score lower than the threshold value of 0.79, corresponding to a q-value of 0.05 was considered to indicate significance.

### Construction of the PPI network combined with the KEGG pathway

For the enriched KEGG pathways, the integrated PPI combined with the KEGG pathways was constructed via EnrichNet, based on the PPI network of the DEGs. Briefly, the PPI network was presented via Cytoscape (http://cytoscape.org/) (15) and then integrated with the PPI associated with the significant KEGG pathways, exhibiting the distribution and mutual connection association of the significant KEGG pathways in the integrated PPI network.

### Protein complexes predicted via ClusterONE

ClusterONE is a graph-clustering algorithm, which is used for forecasting the potential protein complexes in the weighted PPI (16). The weight of the PPI was set as the score provided by STRING.

Subsequently, the predicted protein complexes were verified. A protein complex enriched into a KEGG pathway, a protein domain or a cellular component was identified as a potential protein with function. The protein domain and cellular component were analyzed via the database for annotation, visualization and integrated discovery (17) based on the InterPro (http://www.ebi.ac.uk/interpro/) (18) database and the gene ontology (GO) cellular component to conduct enrichment analysis.

### GO enrichment analysis of the PPI network

GO gene annotation of the PPI network was performed via EnrichNet. GO terms were classified into biological process (BP) and molecular function (MF). The Pearson correlation coefficient was 0.8 and the threshold value for the XD-score was 1.68.

## Results

### Identification of DEGs

To identify the specific DEGs between human FCHL tissues and healthy controls, the publicly available microarray dataset, GSE1010 was obtained from the GEO database. The gene expression profiling data were preprocessed using the Affy package and were normalized by the median method. At P<0.05, a total of 879 DEGs were identified, including 394 upregulated and 485 downregulated genes, of which the 10 most predominant upregulated and downregulated genes are listed in Table I.

### Construction of the PPI network of DEGs

**Figure 1:**
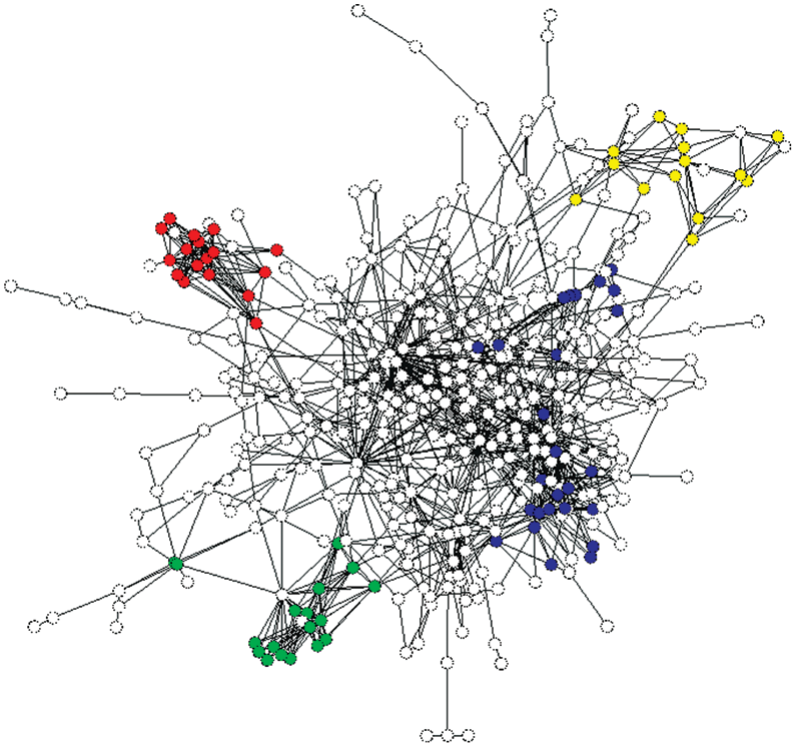
PPI network construction of DEGs combined with KEGG pathway analysis. The colored nodes represent DEGs involved in the four main KEGG pathways. Red indicates hsa00670: One carbon pool by folate, green indicates hsa00592: α-linolenic acid metabolism, blue indicates hsa05310: asthma and yellow indicates hsa00603: Glycosphingolipid biosynthesis-globo series. DEG, differentially expressed genes; PPI, protein-protein interaction; KEGG, Kyoto Encyclopedia of Genes and Genomes.

PPI networks of the DEGs were constructed via STRING. Subsequently, the KEGG pathway enrichment analysis of DEGs in the PPI networks was performed using EnrichNet. The PPI network included 431 nodes and 1,124 associations (Fig. 1). The predominant four KEGG pathways were identified (Table II), including one carbon pool by folate (hsa00670; XD-score=1.064), α-linolenic acid metabolism (hsa00592; XD-score=0.976), asthma (hsa05310; XD-score=0.928), with an XD-score≥079 and glycosphingo-lipid biosynthesis-globo series (hsa00603, XD-score=0.762). The enrichment of the glycosphingolipid biosynthesis pathway may be undervalued by EnrichNet. The DEGs involved in the four main KEGG pathways are in color within the diagram of the PPI network (Fig. 1).

### PPI network of DEGs involved in the KEGG pathway

**Figure 2:**
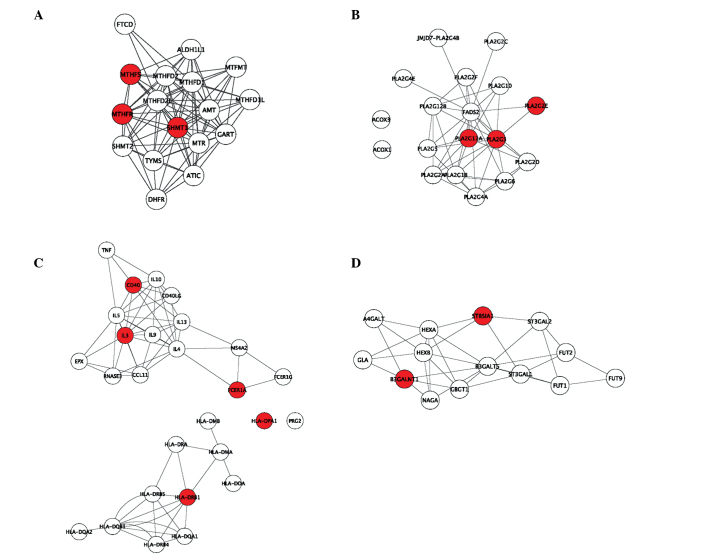
PPI network of DEGs involved in the four main KEGG pathways. (A) Hsa00670, One carbon pool by folate; (B) hsa00592, α-linolenic acid metabolism; (C) hsa05310, asthma; and (D) Hsa00603, glycosphingolipid biosynthesis-globo series. Red nodes indicate the overlapping DEGs between the KEGG pathway and the PPI constructed by the DEGs. DEG, differentially expressed genes; PPI, protein-protein interaction; KEGG, Kyoto Encyclopedia of Genes and Genomes.

**Figure 3:**
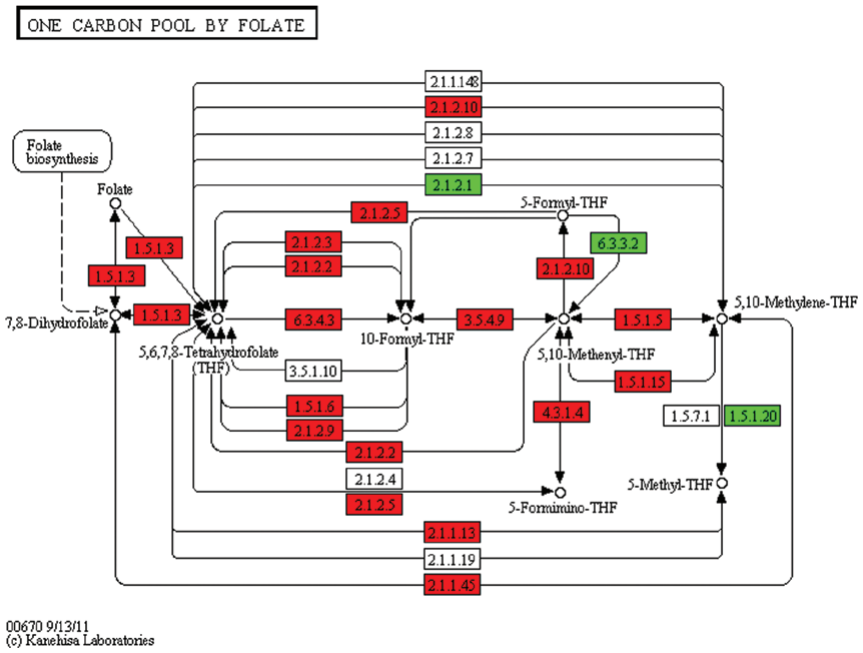
KEGG pathway of hsa00670: one carbon pool by folate. Red indicates the genes involved in the hsa00670 via EnrichNet. Green indicates the overlapping DEGs between the KEGG pathway and the PPI constructed by the DEGs. DEGs, differentially expressed genes; PPI, protein-protein interaction; KEGG, Kyoto Encyclopedia of Genes and Genomes.

The PPI network of DEGs involved in the main four KEGG pathways is shown in Fig. 2, in which the red nodes represent the DEGs. A number of DEGs were located in the center of the PPI network. SHMT1 had a high betweenness value (0.0382) in hsa00670 and it was ranked fifth in all nodes. (Fig. 2A). Additionally, the KEGG pathway of hsa00670 is shown in Fig. 3. SHMT1 encodes serine hydroxymethyltransferase 1, represented as EC 2.1.2.1 (Fig. 3) and is important in this pathway as it catalyzes the hydrolysis of tetrahydrofolate (THF) into 5, 10-methylene-THF. DEGs in other pathways with high betweenness included PLA2G and PLA2G12A in hsa00592 (Fig. 2B), HLA-DRB1 in hsa05310 (Fig. 2C) and B3GALNT1 in hsa00603 (Fig. 2D).

### ClusterONE prediction of protein complexes and its validation

At P<0.01, a total of 10 protein complexes were predicted via ClusterONE (Table III). The genes of Complexes 1–5 exhibited overlap with the four main KEGG pathways, indicating that these genes were differentially enriched in the four KEGG pathways. At the same time, the protein domain classification of DEGs involved in Complexes 1–5 demonstrated that these DEGs were from the same enzyme or signaling molecules and were regulatory in the corresponding KEGG pathway. In addition, the cellular components of these DEGs was consistent.

Complex 6 revealed no KEGG pathway enrichment, but these DEGs were members of the G protein-coupled receptor (GPCR) family, of which 75% were localized in the cell membrane. Approximately 43% of the DEGs of Complex 7 were localized in the synaptic vesicles and the DEGs of Complex 10 were enriched in protein domains without the determined localization. It was difficult to determine whether Complex 7 and 10 may have biological functions. In addition, the function of Complexes 8 and 9 was not verified, which may be due to an error with ClusterONE.

### GO gene annotation of DEGs

GO gene annotation of DEGs revealed 12 BP GO terms (Table IV), including four GO terms associated with bone cell proliferation (GO: 0048762 mesenchymal cell differentiation, GO: 0032331 negative regulation of chondrocyte differentiation, GO: 0045667 regulation of osteoblast differentiation and GO: 0030278 regulation of ossification) and one GO term associated with blood (GO: 0071425 hemopoietic stem cell proliferation). The enriched GO terms associated with bone cells were consistent with the phenomenon that hyperlipidemia causes bone lesions in experimental and clinical settings.

### Discussion

The present study used the EnrichNet online database to analyze RNA samples from patients with FCHL. Initially, the PPI network was constructed for DEGs, subsequently PPI and KEGG pathways were compared in the database (or GO gene annotation) and the KEGG pathway enrichment in DEGs (or GO terms) were identified. KEGG pathway analysis identified four important KEGG pathways, including one carbon pool by folate (hsa00670), α-linolenic acid metabolism (hsa00592), asthma (hsa05310) and the glycosphingolipid biosynthesis-globo series (hsa00603). The one carbon pool by folate pathway consists of the folic acid synthesis of folate THF biosynthesis and the C1-unit conversion process. THF, a carrier of the one-carbon group, acts as a coenzyme DNA-synthesis of nucleic acid and a lack of THF can lead to anemia (19). The α-linolenic acid metabolism KEGG pathway is associated with fatty acid α-linolenic acid metabolism. α-linolenic acid can reduce cholesterol levels in the blood (20) and alleviate the effect of hyperlipidemia (21,22). The asthma pathway enriched by DEGs of patients with hyperlipidemia may be associated with evidence suggesting that hyperlipidemia may cause asthma-associated complications (23). Glycosphingolipid synthesized via the glycosphingolipid biosynthesis-globo series pathway may accumulate in the artery wall and precipitate, which is an established feature of atherosclerosis (24). When this pathway is inhibited, the cholesterol content in the blood is reduced and the degree of atherosclerosis is alleviated (25). The important effect of glycosphingolipid on hyperlipidemia has been discussed previously (26).

Parhami (27) summarized the effects of hyperlipemia on osteoporosis as several patients with atherosclerosis also suffer from osteoporosis. This review suggests that hyperlipemia is the cause of osteoporosis. Further studies have also discussed the association between pathological changes of bone tissue and hyperlipidemia (28,29). For example, although Complex 6 exhibited no KEGG pathway enrichment, these DEGs were identified as members of the GPCR family, of which 75% were localized in the cell membrane. GPCRs have provided novel opportunities for structure-based drug design strategies targeting this protein family (30).

GO function analysis identified 12 enriched BP terms, of which one term was associated with hematopoiesis and four terms were associated with bone cell differentiation. This finding was in accordance with hyperlipidemia and bone lesions in clinical and experimental settings. To date, clinical trials for the treatment of ischemic heart disease and heart failure using bone marrow cells have rapidly increased (31). Baldán *et al* (32) have demonstrated that diet-induced atherosclerosis is impaired when atherosclerotic-susceptible mice are transplanted with ATP-binding cassette sub-family G member 1 (Abcg1)^−/−^ bone marrow. The demonstration that Abcg1^−/−^ macrophages undergo accelerated apoptosis provides a mechanism to explain the decrease in atherosclerotic lesions. Drechsler *et al* (33) provided evidence that hypercho-lesterolemia-induced neutrophilia is multifactorial and that neutrophils infiltrate arteries primarily during early stages of atherosclerosis, which also supports the present results.

In conclusion, the current study identified 897 DEGs and analyzed their functions. Additionally, bioinformatics methods were used to analyze the overlapping DEGs with known genes of the KEGG pathways. Subsequently, the enriched GO terms of DEGs were analyzed. The present study may provide a basis for improved understanding of FCHL. However, experimental studies are required to confirm these findings.

## Figures and Tables

**Table I:** Identification of differentially expressed genes associated with familial combined hyperlipidemia with P<0.05.

Gene symbol	P-value	Log_2_FC
SSX4B///SSX4	5.06E-05	−0.818
TAS2R10	6.16E-05	−1.004
FBXO2	6.61E-05	−0.357
ASMT	1.16E-04	−0.578
BMP7	1.50E-04	−0.394
PCSK1	1.55E-04	−0.750
PSD3	3.55E-04	−0.392
GTF2H3	5.54E-04	−0.525
BTNL8	5.67E-04	−0.619
VIL1	7.08E-04	−0.592
RBM12B	4.92E-05	0.556
TFF1	1.84E-04	0.470
KANSL1L	1.88E-04	0.651
SOX11	2.13E-04	0.590
UMPS	5.59E-04	0.569
RAP1GAP	6.81E-04	0.380
C2orf83	7.06E-04	0.418
SDC4	7.14E-04	0.181
MAST2	9.48E-04	0.407
AFF2	1.06E-03	0.223

Predominant upregulated and downregulated differentially expressed genes are listed.

**Table II:** Main KEGG pathways of differentially expressed genes.

KEGG pathway	XD-score	Fisher q-value	Gene list^b^
hsa00670	1.064	0.331	MTHFS, MTHFR, SHMT1
hsa00592	0.976	0.331	PLA2G2E, PLA2G3, PLA2G12A
hsa05310	0.928	0.301	FCER1A, HLA-DPA1, HLA-DRB1, IL3, CD40
hsa00603	0.762^a^	0.460	ST8SIA1, B3GALNT1

a Represented hsa00603 KEGG pathway with XD-score <0.79 (threshold value).

b The overlapping DEGS between PPI and KEGG. hsa00670, one carbon pool by folate; hsa00592, α-linolenic acid metabolism; hsa05310, asthma; hsa00603, glycosphingolipid biosynthesis-globo series; PPI, protein-protein interaction; KEGG, Kyoto Encyclopedia of Genes and Genomes.

**Table III:** Predicted protein complexes via ClusterONE.

Rank	Protein domains	Relevant pathway	Cellular component^a^	Gene count	Quality^b^	P-value
1	Phospholipase A2	hsa00592	Extracellular region (63%)	20	0.878	4.84E-08
2	THF dehydrogenase; formyl transferase; SHMT;	hsa00670	Mitochondrion (44%)	18	0.891	1.79E-07
3	Sialyltransferase; GTF, family 31; GTF, family 11; GH, family 20	hsa00603	Golgi apparatus (76%)	17	0.756	2.77E-06
4	Four-helical cytokine, core; IL-4; IL-17; TNF 2; Peroxidases heam-ligand binding site; Toll-IL R MHC class I, α chain, α1 and α2	hsa05310	Extracellular space (52%)	27	0.598	3.72E–06
5	Immunoglobulin C-Type	hsa05310	MHC class II protein complex (100%)	9	0.793	5.98E-04
6	GPCR, rhodopsin-like superfamily	NA	Integral to plasma membrane (75%)	12	0.578	7.86E-04
7	NA	NA	Synaptic vesicle (43%)	8	0.701	0.001
8	NA	NA	NA	6	0.645	0.005
9	NA	NA	NA	6	0.625	0.007
10	3′5′-cyclic nucleotide PDE; adenylyl cyclase class-3/4/guanylyl cyclase, conserved site	NA	NA	7	0.711	0.007

a Percentage represents the proportion of cellular component-associated genes in total DEGs used to predict the protein complex.

b The closer the value is to 1, the higher the possibility of a predicted compound. THF, tetrahydrofolate; SHMT, serine hydroxymethyltransferase; GTF, glycosyl transferase; GH, Glycoside hydrolase; IL, interleukin; TNF, tumor necrosis factor; R, receptor; PDE, phosphodiesterase.

**Table IV:** GO gene annotation of differentially expressed genes via EnrichNet.

GO (biological process)	XD-score	Fisher q-value	Gene count
GO:0048762 (mesenchymal cell differentiation)	4.033	0.018	5
GO:0032331 (negative regulation of chondrocyte differentiation)	2.303	0.162	4
GO:0045885 (positive regulation of survival gene product expression)	2.303	0.162	4
GO:0060445 (branching involved in salivary gland morphogenesis)	2.303	0.162	4
GO:0007567 (parturition)	2.233	0.280	3
GO:0090009 (primitive streak formation)	2.233	0.280	3
GO:0060740 (prostate gland epithelium morphogenesis)	2.233	0.280	3
GO:0045667 (regulation of osteoblast differentiation)	2.105	0.194	3
GO:0030278 (regulation of ossification)	1.988	0.317	3
GO:0010039 (response to iron ion)	1.933	0.208	3
GO:0071425 (hemopoietic stem cell proliferation)	1.783	0.332	3
GO:0018298 (protein-chromophore linkage)	1.783	0.332	3
